# First Brazilian patient undergoing nocturnal self-care home hemodialysis

**DOI:** 10.1016/j.clinsp.2025.100681

**Published:** 2025-06-02

**Authors:** Rafael C. S. Tavares, Erica Adelina Guimarães, Rodrigo Dias, Ísisde S.F. Cordeiro, Benedito Jorge Pereira, Rosilene M. Elias

**Affiliations:** a Department of Medicine, Division of Nephrology, Hospital das Clínicas, Universidade de São Paulo, São Paulo, SP, Brazil; b NefroStar Kidney Care, São Paulo, SP, Brazil

Nocturnal home hemodialysis represents a significant advance in the treatment of patients with advanced chronic kidney disease on maintenance hemodialysis since it provides daily flexibility, and improves quality of life, cardiovascular outcomes, and phosphorus control.^1^ Despite these benefits, home hemodialysis remains underutilized in many countries.

There are several reasons that explain the low adoption of home dialysis globally, which may be related to the healthcare system (e.g., preferential policies for home dialysis, relative delivery costs, reimbursement for dialysis in units versus home dialysis), clinics/units (e.g., clinicians' bias against home dialysis, education and specialization of healthcare professionals, reimbursement to patients for dialysis-related costs), and patients (e.g., suitable home environment, physical and cognitive limitations, health literacy).^2^ The *Kidney Disease Improving Global Outcomes* (KDIGO) Controversies Conference on home dialysis, held in 2021, recommended the alignment of policies, resources at the center level, and clear leadership from well-informed and motivated clinical teams to facilitate greater access to home dialysis.^3^

In Brazil, most patients on renal replacement therapy are on conventional hemodialysis in Hospitals or satellite clinics.^4^ A specific aspect of the situation in Brazil is the source of funding for dialysis, where 80 % of patients are covered by the Public Health System, which has limited resources, and there is still no regulation to include patients in Home Hemodialysis (HH).

Here we presented the experience of the first documented Brazilian patient to undergo nocturnal self-care home hemodialysis, highlighting the potential benefits and the challenges encountered in implementing this modality in Brazil.

A young patient with kidney disease secondary to obstructive uropathy who was on conventional hemodialysis since 2016 was invited to initiate an HH training program.

The patient was evaluated by a multidisciplinary team, including physicians, nurses, psychologists, and a social worker. The patient's home conditions were assessed by a specialized technical team to ensure proper storage of dialysate, sufficient space for the hemodialysis machine (Fresenius 4008), and the portable reverse osmosis unit (Vexter). Water treatment and dialysis machine-related issues were covered by a specialized team that was responsible for preventive maintenance and water testing according to the governmental requirement (RDC ANVISA 11 de 13/03/2014).

Between September and December 2024, the patient was intensively trained during hemodialysis in the clinic. A room was specifically selected for this goal and an expert nephrology nurse was designed for this task. The patient was trained in dialysis-related procedures, including preparing the equipment, operating the machine (turning it on and off), understanding and resolving various machine alarms, learning safety protocols, performing fistula cannulation, and managing waste generated after therapy. Each training session focused on a specific topic, which could be revisited in subsequent sessions based on the evaluation by the physician and nurse or upon the patient's request.

Topics that are also addressed during the training included, but were not limited to: education on Arteriovenous Fistula (AVF) care, including infection detection, thrombosis prevention, and cannulation techniques; Hygiene; Dialysis system setup – including ultrafiltration, temperature, blood and dialysate flows, bicarbonate concentration, and sodium; Connecting the AVF to the dialysis circuit; Monitoring of blood pressure before and after each session; Simulation exercises for managing complications such as hypotension, hematomas, and machine alarms; Water treatment – setting up the portable reverse osmosis, performing chloride test before each dialysis session; Waste Disposal: Contaminated vs. Non-Contaminated Materials.

At the end of the training, the patient underwent a theoretical evaluation with questions related to the training material. Additionally, the patient was individually assessed by the multidisciplinary team on the topics covered and their ability to manage safety issues related to the method. The patient was also supervised from a distance while performing tasks independently. The nurse intervened only when necessary for safety or at the patient’s request.

When the patient performed the first dialysis at home, the session was supervised from the beginning to the end by the team. During the first week, the hemodialysis sessions were telemonitored. Furthermore, there was continuous communication between the patient and the team every day.

To empower growing autonomy, during the first month, the patient initiates sessions without direct assistance, under remote supervision if requested. He had full independence in system setup and AVF cannulation. By his own decision, the patient initiated hemodialysis in the afternoon, ensuring each session ended before bedtime for at least 10-days. Afterward, he gradually pushed the start time later and is now performing dialysis overnight. His prescription includes a 6-hour session, 5-days a week, with Sundays and one weekday off. Blood flow is set at 230 mL/min, and dialysate flows at 300 L/min.

The patient was asked to record all blood pressure measurements, pre- and post-dialysis weight, daily ultrafiltration, and any symptoms. Once a month, he returns to in-center dialysis for a review of all records, laboratory exams, and a medical consultation. Adjustments to the dialysis prescription and medications are made monthly, based on the laboratory results.

So far, he is doing well, with no adverse events and an improvement in bone and mineral markers was observed (Table 1). Fig. 1 illustrates to the entire process of training and implementing the home hemodialysis program.

**Table 1:** Laboratory parameters during the first month of self-care hemodialysis.

	Before Home HD	First Week	First Month
**Ionized calcium (mg/dL)**	4.98	4.93	4.58
**Phosphate (mg/dL)**	8.1	7.2	4.7
**Alkaline phosphatase (U/L)**	63	70	69
**Parathyroid hormone (pg/mL)**	232	499	132
**Hemoglobin g/dL**	12.8	13.7	11.0
**Ferritin (ng/mL)**	349.4	128.9	233.0
**Creatinine (mg/dL)**	16.0	8.3	9.7
**Urea (mg/dL)**	129	93	88
**Sodium (mmoL/L)**	138	142	140
**Potassium (mmoL/L)**	5.5	4.8	4.8
**Magnesium (mg/dL)**	3.3	2.5	2.7
**pH**	7.32	7.36	7.35
**Bicarbonate (mEq/L)**	17.5	22.0	19.9

**Fig. 1:**
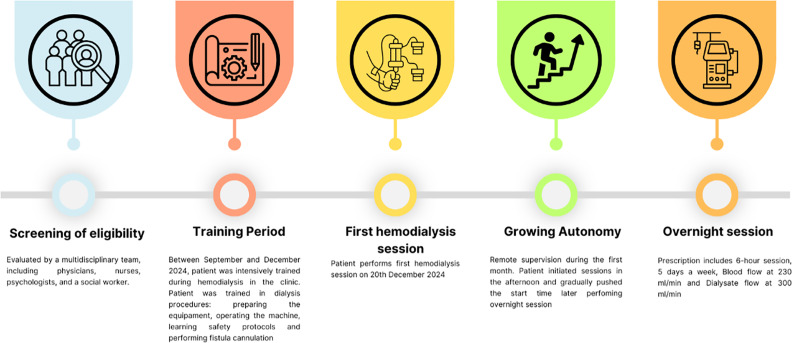
Home hemodialysis program.

This patient's experience is groundbreaking in Brazil and demonstrates that nocturnal self-care home hemodialysis is feasible. Given that most patients on hemodialysis in Brazil rely on the public healthcare system (SUS),^4^ which faces challenges related to access and the availability of in-center hemodialysis slots, home hemodialysis could serve as an alternative solution to this issue. Additionally, as shown in other countries, it may offer a renal replacement therapy option with potential clinical benefits for selected patient groups.

Despite the challenges faced in implementing the home hemodialysis program, this case report highlights a pioneering effort in Brazil to introduce self-care home hemodialysis. It represents a hopeful step forward and aligns with the International Home Dialysis Consortium's initiative to promote equity and expand access to home dialysis worldwide.^5^

This case represents a significant advancement in Brazilian nephrology, establishing a new milestone for self-care hemodialysis. The experience suggests that well-selected and trained patients can benefit from this model, with the potential for large-scale implementation of a novel home-hemodialysis program in Brazil.

## Statement of ethics

Study approval statement: This study protocol was reviewed and approved by Comissão de Análises de Projetos de Pesquisa (Cappesq, Hospital das Clinicas, Universidade de Sao Paulo), approval number 61,016,922.5.0000.0068.

Consent to publish statement: Written informed consent was obtained from the participant.

## Data availability

The data for this case is entirely reported in the text. If the authors require additional information, it can be provided upon reasonable request, while respecting the privacy of personal information.

## Authors’ contributions

Conceptualization and design: RCST, RME. Acquisition of data: RCST, EAG. Analysis and interpretation: RCST, RME. Drafting the article: RCST, RME. Revising: RCST, RD, ISFC, BJP, RME. Approval of the final version RCST, RD, ISFC, BJP, RME. Supervision and project administration: RME.

## Declaration of competing interest

The authors declare no conflicts of interest.
